# Fitness Ranking of Individual Mutants Drives Patterns of Epistatic Interactions in HIV-1

**DOI:** 10.1371/journal.pone.0018375

**Published:** 2011-03-31

**Authors:** Javier P. Martínez, Gennady Bocharov, Anna Ignatovich, Jochen Reiter, Matthias T. Dittmar, Simon Wain-Hobson, Andreas Meyerhans

**Affiliations:** 1 Department of Virology, University of the Saarland, Homburg, Germany; 2 Institute of Numerical Mathematics, Russian Academy of Sciences, Moscow, Russian Federation; 3 Centre for Immunology and Infectious Disease, Barts and The London School of Medicine and Dentistry, London, United Kingdom; 4 Molecular Retrovirology Unit, Institute Pasteur, Paris, France; 5 ICREA Infection Biology Laboratory, Department of Experimental and Health Sciences, University Pompeu Fabra, Barcelona, Spain; Institute of Infectious Disease and Molecular Medicine, South Africa

## Abstract

Fitness interactions between mutations, referred to as epistasis, can strongly impact evolution. For RNA viruses and retroviruses with their high mutation rates, epistasis may be particularly important to overcome fitness losses due to the accumulation of deleterious mutations and thus could influence the frequency of mutants in a viral population. As human immunodeficiency virus type 1 (HIV-1) resistance to azidothymidine (AZT) requires selection of sequential mutations, it is a good system to study the impact of epistasis. Here we present a thorough analysis of a classical AZT-resistance pathway (the 41–215 cluster) of HIV-1 variants by fitness measurements in single round infection assays covering physiological drug concentrations *ex vivo*. The sign and value of epistasis varied and did not predict the epistatic effect on the mutant frequency. This complex behavior is explained by the fitness ranking of the variants that strongly depends on environmental factors, i.e., the presence and absence of drugs and the host cells used. Although some interactions compensate fitness losses, the observed small effect on the relative mutant frequencies suggests that epistasis might be inefficient as a buffering mechanism for fitness losses *in vivo*. While the use of epistasis-based hypotheses to make general assumptions on the evolutionary dynamics of viral populations is appealing, our data caution their interpretation without further knowledge on the characteristics of the viral mutant spectrum under different environmental conditions.

## Introduction

Epistasis, the fitness interaction between mutations, has been suggested to influence the evolutionary dynamics of virus populations [1]. In its original definition by Bateson, epistasis described a phenomenon in which a discrete phenotype derived from a genetic variant in one locus was altered by a genetic variant at another locus [2]. Thus both loci must be located within the same phenotype-determining gene clusters and show some kind of interaction. With this mechanism in mind, epistasis is nowadays commonly used to describe any deviation from an expected phenotype that originates from a combination of mutations.

Fitness interactions can act in different directions. If mutations interact such that their combined effect on fitness is greater than expected from their individual effects, then epistasis is said to be synergistic. By contrast, if mutations interact so that their combined effect is smaller than expected, then epistasis is called antagonistic. Depending on the nature of the mutations being deleterious or beneficial, the sign of epistasis can be positive or negative. When mutations are deleterious, synergistic interactions result in negative epistasis and antagonistic interactions result in positive epistasis. The contrary is true for beneficial mutations were synergistic interactions result in positive epistasis and antagonistic interactions result in negative epistasis [3], [4].

Epistatic effects have been observed in many fundamental biological processes like speciation [5], long-term selection in model organisms [6], and in loci associated with human diseases [7]–[9]. Epistatic interactions also have a central role in evolutionary topics such as the evolution of sexual reproduction [10], [11] and the structural evolution of genetic systems [12], [13]. With respect to viruses, interactions between mutations are also common. Epistasis among viral loci has been found in many diverse viruses like DNA bacteriophage Phi-X174 [14], RNA bacteriophage Phi-6 [15], foot-and-mouth disease virus [16], polio virus [17], vesicular stomatitis virus [18], chikungunya virus [19] and human immunodeficiency virus (HIV) [20]–[24]. Although a wide range of interactions (positive and negative) was observed, a predominance for positive epistasis seems to be the trend which may directly impact viral robustness and the efficiency of variant selection under therapy [1], [3], [18], [25], [26]

HIV is an ideal candidate to study epistatic interactions in viruses. First, the virus shows a phenomenal genetic diversity which is observed at all possible levels from between patient comparisons to variants even within multi-infected individual cells [27]. This genetic diversity is accompanied by the capacity of HIV to rapidly adapt to changing environments. It is best exemplified by the rapid selection of escape mutants in response to antiviral drugs. Depending on the nature of the drug-HIV interaction, it may require just few weeks to fix the resistant mutants within the viral population *in vivo* [28]. Second, HIV grows easily in cell culture and assays to determine viral fitness are well established. Third, there is ample data on HIV sequences and characteristics within patients that allow estimating the consequences of epistatic interactions within infected hosts.

Under the conditions of mutation-selection equilibrium, fitness interactions between mutations will affect the frequency of individual mutants within a virus population. A buffering effect of deleterious mutations is expected if epistasis would be positive. In this case, the abundance of deleterious mutants would be higher than expected and the viral population may respond faster to a new selection pressure supposing that the deleterious mutations would be beneficial under the new growth conditions. A clinically important example and test scenario for this is the selection of drug-resistant HIV variants after the use of antiretroviral treatment. By using amino acid sequence data of the reverse transcriptase and protease regions of HIV-1 isolates from infected individuals undergoing antiviral treatment, and the corresponding fitness values measured *in vitro* in the absence of drugs, statistical evidence for the predominance of positive epistasis has been detected [20]. However, a number of limitations of this study have been raised. One major concern was the likely under-representation of low fit variants in the data set that could have led to false conclusions towards epistasis [29]. Such a biased genome representation seems inevitably linked to the experimental procedure used to generate the genotype to phenotype correlations. A preference for the major viral mutants and thus more fit variants is simply obtained by PCR-mediated amplification and the direct cloning of the respective HIV regions into HIV vectors for subsequent fitness measurements. Thereby the clonal sequence representation is expected to be directly proportional to the respective fitness of the variant *in vivo*. Other limitations of the study were (i) the lack of fitness values in the presence of drugs so that epistatic effects and their consequences could not be compared with and without medication and (ii) the lack of knowledge of the direct path of mutation accumulation including all intermediate mutants.

A straightforward and complementary approach to measure fitness interactions relies on the construction of HIV variants with all mutations along an evolutionary pathway, then measuring their individual and combined fitness effects, and comparing the results with predictions generated under the null hypothesis of non-epistatic interactions [18]. While this approach is experimentally feasible only for a limited number of variants and thus lacks completeness over all possible HIV mutation pathways, it is of highest resolution and captures all variants including those of very low fitness. Here we report the results of such an analysis of a specific mutation pathway that HIV follows within individuals after treatment with azidothymidine (AZT), a nucleoside-analog reverse transcriptase inhibitor. The overall aim was (i) to generate a complete and physiologically relevant HIV data set with fitness values for all mutants within the mutation pathway, (ii) to determine the influence of environmental factors like host cells and antiviral drug concentrations on the fitness interactions of the mutations and (iii) to derive quantitative estimates of the epistatic effects on the relative frequency distribution of the viral mutants. The determined fitness values match the HIV mutant frequencies observed within patients and can explain the observed mutation pathway. Epistatic interactions depend strongly on the host cell environment and decrease with increasing drug concentrations. The relationship between the value of epistasis and the relative mutant frequency is complex and determined by the fitness ranking of individual mutants.

## Results

### Selection of a HIV-1 mutation pathway for analysis of epistatic interactions

**Figure 1:**
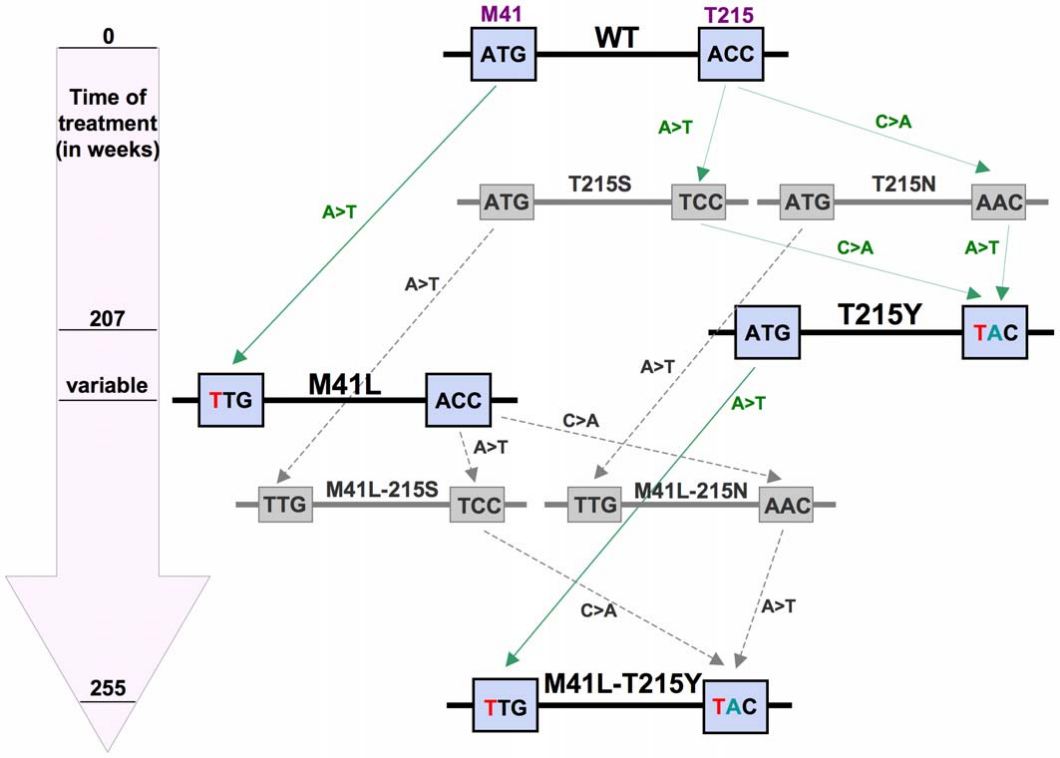
Mutation pathway of the HIV-1 reverse transcriptase under AZT therapy *in vivo*. The scheme shows one common *in vivo* developmental pathway of AZT-resistant HIV-1 mutants at amino acid positions 41 and 215 in the reverse transcriptase. Amino acids are given in the one letter code. In the block arrow, estimated waiting times of mutant appearance are marked. The values are according to estimations from Beerenwinkel, et al. [31] The flowchart arrows highlight the respective nucleotide changes. Mutants found *in vivo* are in bold type while mutants in grey are not observed *in vivo*.

In order to quantify precisely epistatic interactions in HIV and analyze their dependence on environmental factors such as host cells and antiviral drugs, we focused on a specific mutational pathway that HIV-1 follows *in vivo* during treatment with AZT, the prototypic reverse transcriptase (RT) inhibitor first used in infected patients and a common component of current anti-retroviral formulations. When taken up by cells, AZT is phosphorylated by thymidine kinases to the active AZT-triphosphate. Upon incorporation into the nascent HIV DNA strand, RT-dependent chain elongation is stopped due to the 3′azido-group [30]. Treatment of HIV-infected individuals with AZT leads to the selection of AZT-resistant HIV-mutants with defined amino acid changes in the RT. The mechanism of resistance development is well studied [31], [32] and follows specific pathways. One such HIV-1 resistance pathway (Figure 1) is characterized by the key mutations at positions 41 and 215 of the RT [31], [33]. The highly AZT-resistant double mutant M41L-T215Y appears *in vivo* after around 255 weeks of treatment and requires a number of intermediate mutants of which only the M41L and T215Y mutants are commonly observed [31]. However, at least one of the other possible intermediates, T215S, T215N, M41L-T215S and M41L-T215N must have been transiently generated (Figure 1, in grey).

### Generation of AZT-resistant mutants of HIV-1, fitness determination and epistasis calculations

All seven HIV-1 RT mutants from an AZT-resistance pathway (Figure 1) were generated by shuffling PCR. They were then cloned into an HIV-1 NL4-3-based vector that is deficient in the expression of a functional envelope (Env) protein and contains the *Renilla* luciferase gene in the position of *nef* [34]. Respective HIV-1 Env-pseudotyped viruses that can only undergo a single round of infection in susceptible target cells were subsequently produced from 293T cells after co-transfection with RT mutants and an HIV-1 *env* expression plasmid. The relative fitness of the mutant viruses was assessed under a range of physiologically relevant AZT concentrations by infecting the TZM-bl cell-line or primary peripheral blood lymphocytes (PBMCs) from two healthy donors (here referred to as donors 1 and 2) and measuring the relative luciferase activities of the variants compared to that of the wild-type. The epistatic interaction *E* of the mutations was then calculated according to the epistasis definition in a two-locus-two-allele model: *E = W_00_W_11_−W_01_W_10_* [Equation 1], where *W_00_* is the fitness of the wild type, *W_11_* the fitness of the double mutant and *W_01_*, *W_10_* are the fitness of both single mutants, respectively. Observed and expected relative fitness values were then graphically compared as described previously [3], [24].

### The fitness ranking of AZT-resistant HIV-1 RT mutants corresponds to their frequency distribution in AZT-treated patients

**Figure 2:**
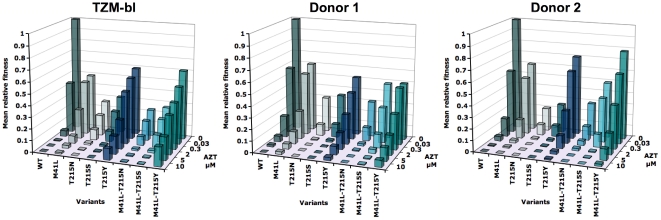
Relative fitness values of HIV-1 reverse transcriptase mutants along an AZT-resistance pathway. Mean fitness values for wild type (WT) and mutants in the TZM-bl cell line and PBMC from Donor 1 and Donor 2 as a function of AZT concentration. The values are the mean of three or four independent infections (the actual values with standard deviations and errors are given in Table S1 and plotted in Figure S1 under supporting information). The mean fitness of the wild type without the addition of drug was set to 1. AZT concentrations range from 0 to 10 µM which cover the physiologically relevant range *in vivo*.

The distribution of fitness relative to that of the wild type for all single and two-point HIV-1 RT mutants infecting TZM-bl cells or PBMC in the presence of 0 to 10 µM AZT is shown in Figure 2 and in Figure S1 and Table S1 under Supporting Information. With the TZM-bl cell line and the PBMC of two blood donors as target cells, the wild-type virus has a varying fitness advantage over the AZT-resistant variants in the absence of drug. Increasing drug concentrations render particularly the mutants M41L, T215Y and M41L/T215Y more fit than the wild type. These dominant RT mutants have been analyzed previously and our determined relative fitness values are concordant with previous findings [35], [36]. Similarly concordant are the inhibitory concentration 50 (IC_50_) values for AZT that have been determined for these mutants [35], [37]–[40] and which can be derived from our fitness measurements as a function of drug concentrations. The newly analyzed intermediate mutants T215S, T215N, M41L-T215S and M41L-T215N of the AZT-resistance pathway (Figure 1) exhibit low fitness values under all drug concentrations with the 215S mutants being slightly fitter than the 215N. Thus, taken together, the ranking order of the fitness values for all RT mutants in the presence of AZT correspond well with the frequency distribution of the respective mutants found in patients under AZT treatment i.e. T215Y > M41L > T215S > T215N (Stanford Drug Resistance Database, http://hivdb.stanford.edu).

The fine structure of the fitness distribution of the RT mutants in TZM-bl and PBMC revealed interesting features that are best visualized in supplementary figure S1. (1) The relative fitness of the AZT-resistant mutants is influenced by the host-cell environment. For example, the mutants T215Y and M41L-T215Y exhibit a higher relative fitness without drug in PBMC of donor 2 than in PBMC of donor 1 or TZM-bl cells. Furthermore, with the exception of the M41L-T215S, the non-dominant RT mutants are fitter in TZM-bl cells under all AZT concentrations than in PBMC. (2) The fitness of the wild type in the presence of AZT is usually higher in PBMC than in TZM-bl cells. This mounts to an about 10-fold and 30-fold difference under 2 µM and 5 µM AZT respectively. Under 10 µM AZT, the replication of the wild type was practically not detectable in all cell-types. (3) The fitness differences between the wild type and the RT mutants are not constant. For example, with 5 µM AZT, the highly resistant mutant M41L/T215Y exhibits a 160-fold higher fitness than the wild type in TZM-bl cells but is only around 6-fold or 20-fold fitter in PBMC of donor 1 and 2, respectively. Thus, the fitness behavior of the wild type and the RT mutants as a function of AZT concentrations is cell type dependent. This correlates well with the observation that HIV replication and adaptation strongly depends on the host-cell environment [41]–[43].

### The HIV-1 AZT-resistance pathway is characterized by strong and varying epistasis between the RT mutations at amino acids 41 and 215

**Figure 3:**
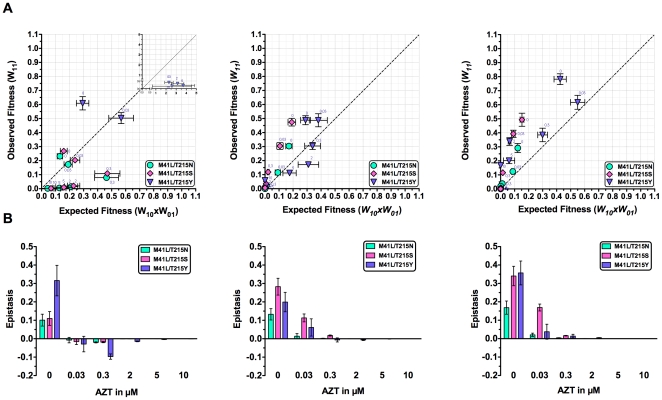
Epistasis in AZT-resistant HIV-1 reverse transcriptase mutants. (A) The observed relative fitness of double mutants W_11_ along an AZT resistance pathway under different AZT concentrations is plotted against the product of the relative fitness of the single mutants W_10_xW_01_. The latter reflect the expected fitness if there is no epistasis. Values are calculated for the fitness in TZM-bl cells and PBMC from two healthy donors (named Donors 1 and 2). The diagonal line corresponds to absence of epistasis while the areas of positive and negative epistasis are above and below respectively. Error bars indicate standard error of the mean. (B) Positive epistasis declines in strength with increasing AZT concentrations. Epistasis values for the three HIV-1 double mutants along the AZT resistance pathway are plotted against AZT concentrations. Error bars indicate standard error of the mean.

In order to analyze possible interactions between the mutations of the key amino acids along the AZT-resistance pathway, we calculated epistasis (*E*) according to equation 1 from the determined fitness values. Without drug pressure, *E* is always strongly positive for the TZM-bl cells and both PBMC Donors (Table S2, under Supporting Information) however the relative values for the three double mutants differ in the target cells. To better visualize the epistatic interactions between all RT mutations, the experimental fitness values of the double mutants (i.e. the observed fitness) were plotted against the products of the fitness of the one-point mutants (i.e. the expected fitness under the assumption of no epistatic interactions) (Figure 3A). Positive epistasis means that the fitness of the double mutants is higher than expected (the diagonal line corresponds to no epistatic effects). To test whether the overall finding of epistatic interactions is statistically robust, we performed bootstrapping to generate randomized data sets (N = 1000) and applied the same analysis. In all cases the mean epistasis values were significantly greater than zero (Table S2 under Supporting Information).

### The epistatic interactions between the mutations changed upon increasing drug pressure and differed between the target cells used

An increase in AZT concentrations resulted in a decrease of epistasis. The three different double mutants showed a varying relative decrease in the different target cells. With TZM-bl cells, the sign of epistasis changed to negative already from the lowest AZT concentration whereas with the PBMC of donors 1 and 2, epistasis was mainly (donor 1) or always (donor 2) positive (Figure 3B). The change in the sign of epistasis in the TZM-bl cells is mainly due to the fitness ranking of the wild type, which is relatively low, and the 1-point mutants, which are relatively high in these cells as compared to PBMC (see Supporting Figure S1). Together these observations show that the type of fitness interaction may change along with the environmental conditions under which it is analyzed. In the absence of drug, the fitness loss due to the acquisition of resistance mutations is compensated by a strong antagonistic interaction (positive epistasis) in all cases. However when AZT is present, the fitness interaction is still antagonistic (now negative epistasis) in TZM-bl cells but becomes mainly synergistic (now positive epistasis) in PBMC. Thus the fitness gain is less than expected for TZM-bl cells but mainly higher than expected for PBMC.

### Epistasis affects the relative abundance of drug-resistant HIV-1 mutants

**Figure 4:**
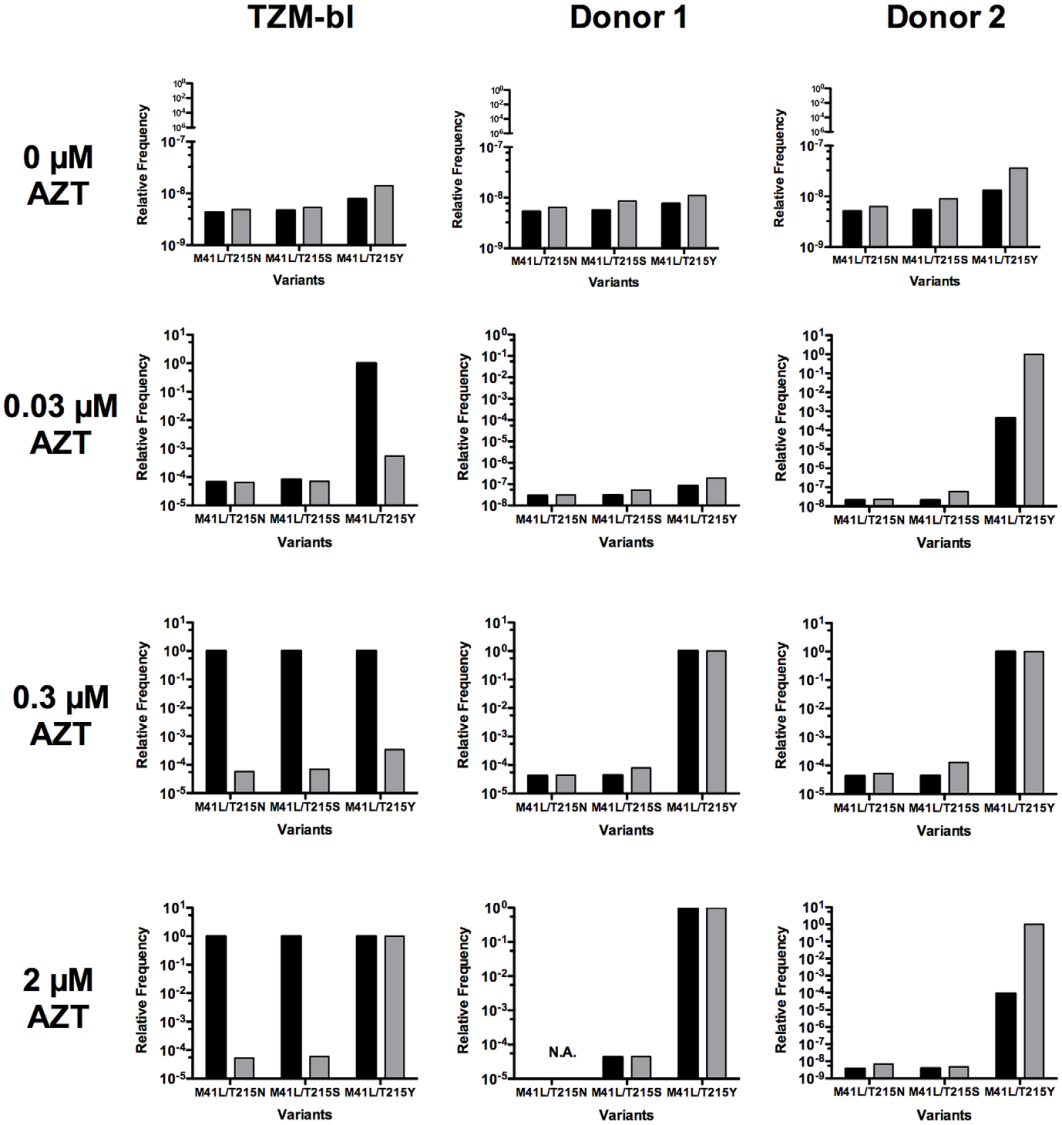
Effect of epistasis on the relative frequencies of drug-resistant HIV-1 mutants. The plot shows the estimated relative frequencies of the double mutants along the AZT resistance pathway under different AZT concentrations for TZM-bl cells and both PBMC donors without epistasis (black bars) and with epistasis (grey bars). Without drug pressure, epistasis has a small effect on the mutant frequencies. With the addtion of drug, epistasis has a varying effect on the mutant frequencies that is dependent on the fitness ranking of the wild type, 1-point mutants and 2-point mutants. Calculations were performed according to an established model specified in equation 2 (see text for details). Fitness values were taken from Table S1.

The fitness interactions between the mutations along a drug-resistance pathway are expected to have an impact on the relative mutant frequencies in a viral population. This in turn may be of great clinical importance for the selection of drug-resistance under antiviral treatment because a higher or lower steady state level of a resistant mutant may lead to a faster or slower outgrowth. Having determined all fitness values for all mutants along the AZT-resistance pathway, we were now able to quantitatively estimate the effect of epistasis on the relative abundance of the double mutants. For this, the expected fitness values under the assumption of no epistasis were calculated from the experimentally determined fitness values and used to analyze the respective steady state frequencies assuming a mutation-selection equilibrium as defined by the general model of HIV quasispecies dynamics [44]. Under this condition, the relative abundance of the wild-type virus and the 1-point and 2-point mutants can be estimated by computing the eigenvectors of the following eigenvalue problem [Equation 2]:
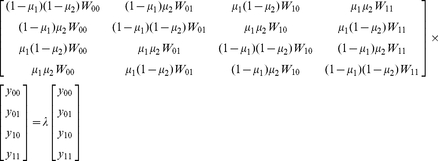



Here, *y_00_* and *y_11_* denote the equilibrium abundance of the wild-type virus and the 2-point mutant, respectively, whereas *y_01_* and *y_10_* denote the equilibrium abundance of the 1-point mutants. The parameters *µ_1_* and *µ_2_* characterize the mutation rate for the first position and for the second position respectively, *λ* is the standard notation for the eigenvalue. Under the simplifying assumption that the mutation rate µ is not affected by the mutations itself, the relative frequencies of the 2-point mutants can be readily calculated using our fitness data (Table S1) and MATLAB routines (www.mathworks.com). The results are shown in Figure 4 and Table S3.

Depending on the AZT concentration, the presence of epistasis has a marginal or a significant effect on the relative frequency distribution of the 2-point mutants within the virus population. Importantly, high epistasis values do not directly predict a large effect on that distribution. For example, without AZT when epistasis is highest, the relative mutant frequencies are around 10^−8^ to 10^−9^ and epistasis affect those frequencies up to 3-fold (Figure 4 and Table S3). In the presence of AZT when epistasis values were significantly smaller, frequency effects of up to around 10^4^-fold are observed. Taking the M41L/T215Y mutant in the presence of 0.03 µM AZT as an example, epistasis increased the relative frequency around 10^4^-fold according to the measurements in PBMC of donor 2 (Table S3c), diminished it around 10^4^-fold according to the measurements in TZM-bl (Table S3a) or left it relatively unchanged (PBMC donor 1, Table S3b). Furthermore, the frequency effects were not linear. Taking again the M41L/T215Y mutant, epistasis increased the relative frequency around 10^4^-fold at 0.03 µM AZT and 2 µM AZT, however had nearly no effect at 0.3 µM AZT. This complex behavior is due to the fact that the epistasis effect on mutant frequencies strongly depends on the distribution of the relative fitness values for wild type, intermediate mutants and the 2-point mutant, i.e. their ranking. If the intermediate one-point mutants (at least one of them) have larger fitness value than the two-point mutant would have in the absence of epistasis then the effect would be strong. Otherwise the two-point mutant will dominate with and without epistasis. Let us consider two examples: Under 0.3 µM AZT in PBMC of donor 2, the fitness values for wild type, M41L, T215Y and M41L-T251Y are 0.162±0.010, 0.166±0.013, 0.293±0.014 and 0.385±0.047, respectively (see Table S1c for all values of the standard errors of the mean and Table S4 for evaluation of the significance of the fitness differences). Thus, one would expect that the M41L-T251Y mutant will dominate because of its highest fitness and its relative frequency is expected to be close to 1 (in fact 0.999; Table S3c). Assuming no epistatic interaction, the relative fitness for the same variants are 0.162±0.010, 0.166±0.013, 0.293±0.014 and 0.300±0.069, respectively. The M41L-T251Y mutant still dominates because it has the highest fitness and its relative frequency is close to 1 (0.998). In the case of 2 µM AZT, the fitness values with epistasis are 0.0426±0.0027, 0.0236±0.0029, 0.116±0.0078 and 0.187±0.010, respectively. The M41L-T251Y mutant will dominate with a relative frequency close to 1 (in fact 0.999). Assuming no epistasis, the relative fitness is 0.0426±0.0027, 0.0236±0.0029, 0.116±0.0078 and 0.0642±0.020, respectively. Now the 1-point T251Y mutant will dominate because it has the highest fitness and its relative frequency is expected to be close to 1. In this case the relative frequency of the two point mutant M41L-T251Y is only 0.0000897. Thus the epistatic effect is large although the epistasis value is small (Figure 3B and Figure 4).

## Discussion

Epistasis is a fundamental component of the genetic architecture of biological entities and has been suggested to influence the evolutionary dynamics of virus populations. Here we have quantified the fitness of all HIV-1 mutants along the classical AZT-resistance pathway under physiological drug concentrations *ex vivo*, calculated the epistasis values and estimated its impact on mutant frequencies within the viral population. Overall, the pattern of epistasis is complex and dependent on the drug concentrations and the host cells used. Without AZT, epistasis is consistently positive within the TZM-bl cell line and the PBMCs of both blood donors, and results in an estimated 1.5 to 2.8-fold increase in the ratio of the highly resistant 2-point mutant M41L-T251Y to wild type. The presence of AZT leads to a concentration-dependent decrease of epistasis. This is due to the fitness decrease of all variants especially the wild type in the presence of AZT and the respective reduction of the difference of their products (see equation 1 and figure 2). Furthermore, the signs and values of epistasis differ depending on the host cells used and the numerical values do not predict the epistatic effect on the mutant frequency. This complex behavior is explained by the fitness ranking of all mutants in the presence of AZT and the uneven fitness distribution of the 1-point mutants.

The observed positive epistasis under drug-free conditions has a buffering effect on the mutant distribution and caused an increase of the relative frequency of the highly AZT-resistant 2-point mutant M41L-T251Y over the expected frequency if epistasis would be absent. However this relative frequency increase from about 1×10^−8^ to about 3.6×10^−8^ (see Table S3) within the virus population is only marginal considering the published estimates of the HIV effective population size *in vivo* of around 10^3^ to 10^4^ [45]. In the presence of AZT, the sign of epistasis varies with the drug concentrations and the host cells used. As a consequence, epistasis increases or decreases the relative 2-point mutant frequencies or leaves it relatively unchanged. For example, under 0.03 µM AZT and TZM-bl as host cells, epistasis decreases the relative frequency of M41L-T215Y from 1 to 5.5×10^−4^, whereas with PBMC from Donor 2, epistasis increases the relative frequency from 4.5×10^−4^ to almost 1. Although these estimated relative frequencies are now in the range of the effective population size of HIV *in vivo*, a condition where epistatic effects are expected to be relevant, there are no consistent biological criteria to derive general statements on the importance of the epistatic effects for the mutant frequency at the population level.

Epistasis was suggested to contribute to viral robustness, the ability of a virus to maintain stable functioning despite genetic and environmental perturbations [1], [25]. In general, single mutations in a viral genome are deleterious and will reduce viral fitness. The observed predominance of positive epistasis among viral genomes suggests a buffering effect for subsequent mutants such that the mutant spectrum is enlarged [25], [46], [47]. This in turn may become beneficial for the virus population in the context of a strong selection pressure like antiviral treatment. However as we show here for the mutants along the AZT resistance pathway, the buffering effect without AZT is so low that positive epistasis is unlikely to be a major contributing factor to the robustness of HIV i.e. to allow low fitness mutants to survive in an HIV population *in vivo*. Other mechanisms than epistasis may be considered as of prime importance within infected individuals. First, the HIV provirus can persist for months independent of the replicative capacity of the respective mutant [48]. Second, multi-infection of single cells *in vivo* is common and thus phenotypic mixing can contribute to mutant survival [27], [49]. The recent suggestion that HIV is evolving towards a more robust population due to the selection of a lower fitness landscape is compatible with such a scenario [26], [50].

The development of drug resistance in HIV infection remains one of the most challenging difficulties in antiviral treatment. The dynamics of resistant mutants depends on a number of virus replication parameters such as the fitness values, the number of available target cells and mutation/recombination rates. Although the interplay between these factors has been studied using mathematical models, their results suffer from not being based on exact values for all parameters. Fitness is a major determinant of the selection process of drug-resistant mutants that is amenable to experimental quantification. In this respect, the fitness estimates for a complete spectrum of AZT-resistant mutants as a function of the drug concentrations obtained in our work established a solid quantitative basis for further data-driven *in silico* studies.

In summary, our study provides high-resolution fitness values along an important HIV drug-resistance mutation pathway and quantifies the impact of epistasis on mutant frequencies. The pattern of epistatic interactions between the specific mutations is complex and dependent on environmental factors such as the presence and absence of drugs and the host cells used. While some interactions compensate fitness losses, the effect on the relative mutant frequencies was small so that epistasis as a buffering mechanism for fitness losses might be rather inefficient. Together these data caution against over-interpreting qualitative data on epistasis for evolutionary dynamics of viruses without knowledge of the fitness ranking of the complete mutant spectrum.

## Materials and Methods

### Experimental Design

To measure the fitness of HIV-1 RT mutants along an AZT resistance pathway and to evaluate the effect of epistasis ( =  the fitness interactions between the mutations), we generated the respective RT mutants by site-directed mutagenesis PCR [51]. They were then cloned into the HIV-1 viral vector TN7-Stopp that carries the *Renilla* luciferase reporter gene instead of *nef* and lacks a functional *env* gene [34]. HIV pseudotypes were produced by co-transfection of 293T cells with an HIV-1 *env* expression plasmid. The fitness values of the mutants were quantified by measuring the replication capacity of the mutants as the % relative luciferase activity, compared to that of the wild type, after single round infection of target cells with and without the addition of AZT.

### Generation of wild type and drug resistant mutants

#### Mutagenesis

PCR site-directed mutagenesis was used to introduce the desired nucleotide changes at codons 41 and 215 of the reverse transcriptase gene of the HIV-1 NL4-3 molecular clone. This technique involves two PCR reactions using overlapping primers (see Table S5 for primer details). Briefly, in the first PCR, a 1062 base pair fragment containing the desired region was amplified using a 5′ primer carrying a unique restriction site for BclI and the 3′ overlapping primer carrying the desired RT mutation. In parallel, another PCR used the 3′ primer carrying the restriction site for AgeI and the 5′ overlapping primer carrying the other desired RT mutation. The resulting fragments were isolated from an agarose gel and used as templates in the second PCR with only the 5′ BclI and the 3′ AgeI primers giving rise to the final template. The thermocycling conditions for the first PCR were 94°C for 2 min followed by 30 cycles of 94°C 30 s, 58°C 30 s (50°C for the second PCR) and 68°C 45 s (72°C, 1 min for the second PCR) and then a final elongation step of 68°C for 7 min. The introduced restriction sites were used for the subsequent cloning of the mutated fragment.

#### Plasmids and cloning

The PCR products were ligated into the pGEM-T vector (Promega) via TA cloning following manufacturer's instructions. *E. coli* ER2925 (Stratagene) were then transformed and selected on ampicillin agar plates. Plasmid DNA was extracted using the alkaline method. Samples were run on an agarose gel, isolated and sequenced by dideoxy-sequencing. Plasmids containing the desired RT mutants were digested with AgeI and BclI (New England Biolabs) and the respective fragments were inserted into the pTN7-Stopp HIV-1 expression plasmid using the same restriction sites. pTN7-Stopp is derived from the HIV-1 NL4-3 based viral vector and has the luciferase gene in the position of the *nef* gene [34]. It is designed for a single round of replication since it does not express the envelope gene due to the insertion of two nucleotides in the 5′region of the signal peptide resulting in a frameshift and premature termination of translation.

#### Generation of HIV-1 pseudovirus stocks

For pseudo-typed virus generation, wild type or mutated pTN7-Stopp plasmids were co-transfected with a specific plasmid expressing a CCR5-tropic HIV-1 envelope gene into the 293T cell line [52]. Briefly, 293T cells (10^6^ cells) were transfected with 4 µg of each plasmid DNA using Lipofectamine (Invitrogen) according to the manufacturer's instructions. 48 hours after transfection, virus-containing supernatants were harvested, filtered with 0.45 µm cellulose-acetate filters (Schleicher & Schuell), titrated and stored in 1 ml aliquots at −80°C until use.

#### Range of AZT concentrations

We used AZT at final concentrations of 0, 0.03, 0.3, 2, 5 and 10 µM for the infection experiments. These cover the physiological concentrations of anti-retroviral therapy [53], [54].

#### HIV-1 pseudotype infections and determination of relative fitness

Target cells for infection experiments were peripheral blood mononuclear cells (PBMCs) from healthy blood donors and TZM-bl cells. TZM-bl is a HeLa cell line, which expresses CD4 and both CCR5 and CXR4 co-receptors [55], [56]. Cells were plated in triplicate or quadruple on 96 well plates at a density of 10^5^ cells/well and left untreated (0 µM AZT) or pre-incubated for 2 hours with AZT at the final concentrations mentioned above before infections. PBMCs from healthy donors were stimulated overnight with PHA prior to the drug incubation. Infections were performed for each drug treatment by adding wild-type and mutant virus at an MOI of 0,5 in 100 µl of medium to untreated or treated cells. Forty-eight hours after infection, cells were washed and assayed for luciferase activity with the Luciferase Reaction Kit (Promega) according to the manufacturer's instructions. In addition, the relative luciferase activities from infections were normalized to the transfection efficiencies of 293T cells during HIV pseudotype preparation. The final relative fitness values for all mutants were the obtained by relating them to the fitness of the wild type (see below).

#### Statistical analysis

The method of estimating the mean fitness value for every mutant and AZT dose was performed as following: (i) the mean value of a given number of independent measurements of RLU/s was estimated (RLUmutant); (ii) the mean background value, i.e. control (RLUcontrol) was calculated to (iii) correct the original value for the background by subtracting it (RLUcorrected = RLUmutant-RLUcontrol) and then (iv) to normalize the corrected replication capacity using the transfection efficiency parameter, teff, thus getting absolute fitness estimate f_abs_ = RLUcorrected/teff. Finally, the absolute fitness estimates were normalized with respect to the absolute fitness value of the wt genome without drug (f_0_): f = f_abs_/f_0_. The above estimates of the mean of the mutant fitness by using the sample means were supplemented by evaluating their sample variances using the formulas for variances of the products and ratios of independent random variables [57]. The donor cell data were used to estimate the mean fitness and variance values for each mutant using standard formulas for the mean and variance of the sum of independent random variables. To evaluate the epistasis values two different approaches were used. One is based upon a direct calculation of the mean and variance values using the epistasis formula and the rules for dealing with the functions of random variables [57]. The second one implements a parametric bootstrap method to make inference about the mean and its associated standard error. To this end a normal distribution with the corresponding estimated sample mean and variance was used to draw the fitness values of wt, single- and double mutant genomes with the size of the generated sample of 1000. The 95% confidence intervals shown in Tables S1 and S2 under Supporting Information were calculated using the bootstrap estimates of the standard deviation (SD) as mean ±1.96*SD.

## Supporting Information

Figure S1 Bar-plot showing the comparative and high-resolution cell-to-cell fitness distribution of the wild type and RTase 1-point and 2-point mutants along an AZT resistance pathway under different AZT concentrations. Error bars are standard error of the mean.
(TIF)

Table S1 Complete set of the relative fitness values and statistics for the wild type and RTase 1-point and 2-point mutants along an AZT resistance pathway measured under different AZT concentrations in the TZM-bl cell line (a), Donor 1 (b) and Donor 2 (c).
(DOC)

Table S2 The calculated epistasis values and statistics for the RTase 2-point mutants along an AZT resistance pathway under different AZT concentrations in the TZM-bl cell line (a), Donor 1 (b) and Donor 2 (c). Calculations were made according to equation 1 (see main text and materials and methods for details).
(DOC)

Table S3 Relative frequency values for the RTase 2-point mutants along an AZT resistance pathway under different AZT concentrations in the TZM-bl cell line (a), Donor 1 (b) and Donor 2 (c). Relative frequencies with positive epistasis are noted in bold and with negative epistasis in italic. Frequencies were calculated according to equation 2 (see main text for details).
(DOC)

Table S4 Evaluation of statistical significance of the differences in mean relative fitness between wild type and AZT-resistant variants for Donor 2 by a Student-Newman-Keuls test. Fitness values are taken from Table S1c. Calculations were performed in Graphpad Prism. The examples selected here for statistical evaluation are discussed under results.
(DOC)

Table S5 Primers uses for the site-directed PCR to generate the HIV-1 RTase mutants used in this study. Restriction sites are underlined and bold, codons with the introduced mutantions are underlined.
(DOC)
