# Supplementary figures and images for: Fitness Ranking of Individual Mutants Drives Patterns of Epistatic Interactions in HIV-1
Source: PLoS One. 2011 Mar 31;6(3):e18375. doi: 10.1371/journal.pone.0018375 (PMC3069090; doi:10.1371/journal.pone.0018375)

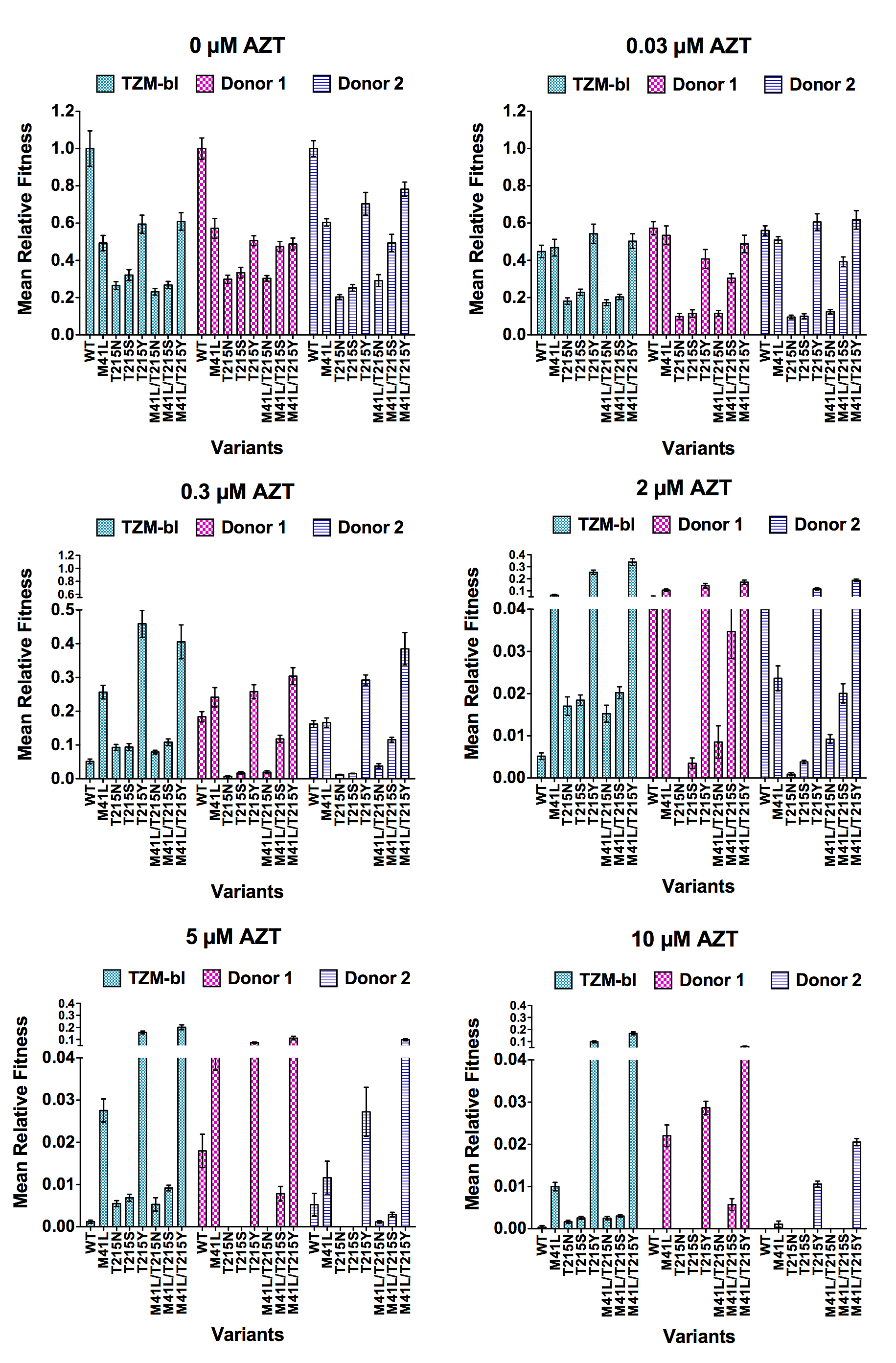

Supplement: Figure S1 — Bar-plot showing the comparative and high-resolution cell-to-cell fitness distribution of the wild type and RTase 1-point and 2-point mutants along an AZT resistance pathway under different AZT concentrations. Error bars are standard error of the mean. (TIF) [file pone.0018375.s001.tif]
