# Supplementary material for: Fitness Ranking of Individual Mutants Drives Patterns of Epistatic Interactions in HIV-1
Source: PLoS One. 2011 Mar 31;6(3):e18375. doi: 10.1371/journal.pone.0018375 (PMC3069090; doi:10.1371/journal.pone.0018375)
Supplement: Table S1 — Complete set of the relative fitness values and statistics for the wild type and RTase 1-point and 2-point mutants along an AZT resistance pathway measured under different AZT concentrations in the TZM-bl cell line (a), Donor 1 (b) and Donor 2 (c). (DOC) [file pone.0018375.s002.doc]

**Table S1a.** Relative fitness values and statistics for the wild-type and AZT-resistant HIV-1 RTase mutants in the TZM-bl cell line

|  |  | **Variants** | | | | | | | |
| --- | --- | --- | --- | --- | --- | --- | --- | --- | --- |
| **AZT (µM)** | **Statistics** | **WT** | **M41L** | **T215N** | **T215S** | **T215Y** | **M41L/T215N** | **M41L/T215S** | **M41L/T215Y** |
| **0** | **mean** | **1** | **0,49293** | **0,26555** | **0,32099** | **0,59467** | **0,23177** | **0,26802** | **0,60889** |
|  | **var** | 0,03634 | 0,0068713 | 0,0017272 | 0,003336 | 0,0092782 | 0,0012855 | 0,0014603 | 0,0089212 |
|  | **std.dev** | 0,19063 | 0,082893 | 0,041559 | 0,057758 | 0,096324 | 0,035854 | 0,038214 | 0,094452 |
|  | **std.err** | 0,095315 | 0,0414465 | 0,0207795 | 0,028879 | 0,048162 | 0,017927 | 0,019107 | 0,047226 |
| **0.03** | **mean** | **0,44741** | **0,46827** | **0,18123** | **0,22827** | **0,54255** | **0,17291** | **0,20357** | **0,50312** |
|  | **var** | 0,0044882 | 0,0079099 | 0,0011664 | 0,0011296 | 0,010668 | 0,001054 | 0,00081364 | 0,0061188 |
|  | **std.dev** | 0,066994 | 0,088938 | 0,034153 | 0,03361 | 0,10328 | 0,032465 | 0,028524 | 0,078223 |
|  | **std.err** | 0,033497 | 0,044469 | 0,0170765 | 0,016805 | 0,05164 | 0,0162325 | 0,014262 | 0,0391115 |
| **0.3** | **mean** | **0,051425** | **0,25668** | **0,092919** | **0,094054** | **0,45925** | **0,07909** | **0,10834** | **0,40557** |
|  | **var** | 0,00019759 | 0,0016176 | 0,0003145 | 0,00040375 | 0,0065834 | 0,00012109 | 0,00037954 | 0,01002 |
|  | **std.dev** | 0,014057 | 0,040219 | 0,017734 | 0,020093 | 0,081138 | 0,011004 | 0,019482 | 0,1001 |
|  | **std.err** | 0,0070285 | 0,0201095 | 0,008867 | 0,0100465 | 0,040569 | 0,005502 | 0,009741 | 0,05005 |
| **2** | **mean** | **0,0051893** | **0,064249** | **0,017045** | **0,018443** | **0,25434** | **0,015259** | **0,020222** | **0,33798** |
|  | **var** | 2,51E-06 | 0,00016126 | 1,92E-05 | 6,20E-06 | 0,0013022 | 1,61E-05 | 7,88E-06 | 0,0030183 |
|  | **std.dev** | 0,0015848 | 0,012699 | 0,0043764 | 0,00249 | 0,036086 | 0,0040139 | 0,002807 | 0,054939 |
|  | **std.err** | 0,0007924 | 0,0063495 | 0,0021882 | 0,001245 | 0,018043 | 0,00200695 | 0,0014035 | 0,0274695 |
| **5** | **mean** | **0,001186** | **0,027546** | **0,0054781** | **0,0068521** | **0,15889** | **0,0052818** | **0,0091683** | **0,20133** |
|  | **var** | 6,11E-07 | 2,95E-05 | 1,99E-06 | 2,77E-06 | 0,00050966 | 1,00E-05 | 1,76E-06 | 0,0016762 |
|  | **std.dev** | 0,00078162 | 0,0054343 | 0,0014115 | 0,0016658 | 0,022576 | 0,0031688 | 0,0013262 | 0,040942 |
|  | **std.err** | 0,00039081 | 0,00271715 | 0,00070575 | 0,0008329 | 0,011288 | 0,0015844 | 0,0006631 | 0,020471 |
| **10** | **mean** | **0,00037756** | **0,0099972** | **0,0016468** | **0,0025467** | **0,097839** | **0,0024935** | **0,0030081** | **0,16804** |
|  | **var** | 5,80E-07 | 4,01E-06 | 5,53E-07 | 5,26E-07 | 0,00028192 | 6,91E-07 | 3,20E-07 | 0,00057946 |
|  | **std.dev** | 0,0007618 | 0,0020014 | 0,00074341 | 0,00072515 | 0,016791 | 0,0008314 | 0,00056569 | 0,024072 |
|  | **std.err** | 0,0003809 | 0,0010007 | 0,000371705 | 0,000362575 | 0,0083955 | 0,0004157 | 0,000282845 | 0,012036 |

**Table S1b.** Relative fitness values and statistics for the wild-type and AZT-resistant HIV-1 RTase mutants in PBMC from Donor 1

|  |  | **Variants** | | | | | | | |
| --- | --- | --- | --- | --- | --- | --- | --- | --- | --- |
| **AZT (µM)** | **Statistics** | **WT** | **M41L** | **T215N** | **T215S** | **T215Y** | **M41L/T215N** | **M41L/T215S** | **M41L/T215Y** |
| **0** | **mean** | **1** | **0,57193** | **0,29873** | **0,33441** | **0,50579** | **0,30354** | **0,47412** | **0,48842** |
|  | **var** | 0,013094 | 0,011296 | 0,0019313 | 0,003367 | 0,0028137 | 0,00090975 | 0,0029509 | 0,0040698 |
|  | **std.dev** | 0,11443 | 0,10628 | 0,043947 | 0,058025 | 0,053044 | 0,030162 | 0,054322 | 0,063795 |
|  | **std.err** | 0,057215 | 0,05314 | 0,0219735 | 0,0290125 | 0,026522 | 0,015081 | 0,027161 | 0,0318975 |
| **0.03** | **mean** | **0,57248** | **0,5343** | **0,097951** | **0,11452** | **0,40772** | **0,11551** | **0,30437** | **0,4876** |
|  | **var** | 0,0049237 | 0,010118 | 0,0010892 | 0,0016226 | 0,010228 | 0,00093316 | 0,0023029 | 0,0086912 |
|  | **std.dev** | 0,070169 | 0,10059 | 0,033003 | 0,040281 | 0,10113 | 0,030548 | 0,047989 | 0,093226 |
|  | **std.err** | 0,0350845 | 0,050295 | 0,0165015 | 0,0201405 | 0,050565 | 0,015274 | 0,0239945 | 0,046613 |
| **0.3** | **mean** | **0,1836** | **0,24162** | **0,0072649** | **0,017038** | **0,25798** | **0,019572** | **0,11819** | **0,30422** |
|  | **var** | 0,00092875 | 0,0032814 | 1,59E-05 | 8,43E-05 | 0,0016884 | 7,82E-05 | 0,00045846 | 0,0025129 |
|  | **std.dev** | 0,030475 | 0,057283 | 0,0039862 | 0,0091832 | 0,04109 | 0,0088438 | 0,021412 | 0,050128 |
|  | **std.err** | 0,0152375 | 0,0286415 | 0,0019931 | 0,0045916 | 0,020545 | 0,0044219 | 0,010706 | 0,025064 |
| **2** | **mean** | **0,048489** | **0,10539** | **0** | **0,0034896** | **0,14252** | **0,0085322** | **0,034745** | **0,17162** |
|  | **var** | 0,00029066 | 0,00034245 |  | 6,47E-06 | 0,0013833 | 5,92E-05 | 0,00016548 | 0,0013456 |
|  | **std.dev** | 0,017049 | 0,018505 |  | 0,0025442 | 0,037192 | 0,0076925 | 0,012864 | 0,036683 |
|  | **std.err** | 0,0085245 | 0,0092525 |  | 0,0012721 | 0,018596 | 0,00384625 | 0,006432 | 0,0183415 |
| **5** | **mean** | **0,017957** | **0,042661** | **0** | **0** | **0,073836** | **0** | **0,0078361** | **0,11186** |
|  | **var** | 6,25E-05 | 0,00012238 |  |  | 0,00015948 |  | 1,18E-05 | 0,00089363 |
|  | **std.dev** | 0,007907 | 0,011063 |  |  | 0,012629 |  | 0,0034401 | 0,029894 |
|  | **std.err** | 0,0039535 | 0,0055315 |  |  | 0,0063145 |  | 0,00172005 | 0,014947 |
| **10** | **mean** | **0** | **0,022027** | **0** | **0** | **0,028658** | **0** | **0,0057119** | **0,059252** |
|  | **var** |  | 2,65E-05 |  |  | 9,88E-06 |  | 7,96E-06 | 3,42E-05 |
|  | **std.dev** |  | 0,0051437 |  |  | 0,003143 |  | 0,0028207 | 0,0058499 |
|  | **std.err** |  | 0,00257185 |  |  | 0,0015715 |  | 0,00141035 | 0,00292495 |

**Table S1c.** Relative fitness values and statistics for the wild-type and AZT-resistant HIV-1 RTase mutants in PBMC from Donor 2

|  |  | **Variants** | | | | | | | |
| --- | --- | --- | --- | --- | --- | --- | --- | --- | --- |
| **AZT (µM)** | **Statistics** | **WT** | **M41L** | **T215N** | **T215S** | **T215Y** | **M41L/T215N** | **M41L/T215S** | **M41L/T215Y** |
| **0** | **mean** | **1** | **0,60398** | **0,20344** | **0,25286** | **0,70386** | **0,29144** | **0,49304** | **0,78211** |
|  | **var** | 0,0073411 | 0,0015884 | 0,00064431 | 0,0012505 | 0,014985 | 0,0042076 | 0,0086832 | 0,0059639 |
|  | **std.dev** | 0,08568 | 0,039855 | 0,025383 | 0,035363 | 0,12241 | 0,064866 | 0,093184 | 0,077226 |
|  | **std.err** | 0,04284 | 0,0199275 | 0,0126915 | 0,0176815 | 0,061205 | 0,032433 | 0,046592 | 0,038613 |
| **0.03** | **mean** | **0,56022** | **0,50883** | **0,095828** | **0,10023** | **0,60567** | **0,12426** | **0,39282** | **0,61736** |
|  | **var** | 0,0025148 | 0,00131 | 0,00040334 | 0,00069277 | 0,0077787 | 0,00056515 | 0,002638 | 0,0099144 |
|  | **std.dev** | 0,050148 | 0,036194 | 0,020083 | 0,026321 | 0,088197 | 0,023773 | 0,051362 | 0,099571 |
|  | **std.err** | 0,025074 | 0,018097 | 0,0100415 | 0,0131605 | 0,0440985 | 0,0118865 | 0,025681 | 0,0497855 |
| **0.3** | **mean** | **0,16226** | **0,16627** | **0,011927** | **0,01554** | **0,29251** | **0,038069** | **0,11565** | **0,38495** |
|  | **var** | 0,00041961 | 0,00075498 | 8,14E-06 | 1,91E-06 | 0,00089438 | 1,85E-04 | 0,00020138 | 0,0091974 |
|  | **std.dev** | 0,020485 | 0,027477 | 0,0028524 | 0,0013832 | 0,029906 | 0,013586 | 0,014191 | 0,095903 |
|  | **std.err** | 0,0102425 | 0,0137385 | 0,0014262 | 0,0006916 | 0,014953 | 0,006793 | 0,0070955 | 0,0479515 |
| **2** | **mean** | **0,042648** | **0,023637** | **0,00092379** | **0,0038235** | **0,11608** | **0,020068** | **0,00923** | **0,18732** |
|  | **var** | 3,00E-05 | 3,45E-05 | 5,80E-07 | 5,17E-07 | 0,00024885 | 2,07E-05 | 4,53E-06 | 0,00040731 |
|  | **std.dev** | 0,0054749 | 0,0058777 | 0,00076155 | 0,00071894 | 0,015775 | 0,0045548 | 0,002129 | 0,020182 |
|  | **std.err** | 0,00273745 | 0,00293885 | 0,000380775 | 0,00035947 | 0,0078875 | 0,0022774 | 0,0010645 | 0,010091 |
| **5** | **mean** | **0,0052242** | **0,011629** | **0** | **0** | **0,02723** | **0,0011425** | **0,0029013** | **0,097714** |
|  | **var** | 2,85E-05 | 6,11E-05 |  |  | 0,00013455 | 3,09E-07 | 1,18E-06 | 0,00029818 |
|  | **std.dev** | 0,0053342 | 0,0078171 |  |  | 0,011599 | 0,00055551 | 0,0010847 | 0,017268 |
|  | **std.err** | 0,0026671 | 0,00390855 |  |  | 0,0057995 | 0,000277755 | 0,00054235 | 0,008634 |
| **10** | **mean** | **0** | **0,0010917** | **0** | **0** | **0,010589** | **0** | **0** | **0,020544** |
|  | **var** |  | 2,10E-06 |  |  | 2,12E-06 |  |  | 2,85E-06 |
|  | **std.dev** |  | 0,0014494 |  |  | 0,0014559 |  |  | 0,0016868 |
|  | **std.err** |  | 0,0007247 |  |  | 0,00072795 |  |  | 0,0008434 |
