# Supplementary material for: Fitness Ranking of Individual Mutants Drives Patterns of Epistatic Interactions in HIV-1
Source: PLoS One. 2011 Mar 31;6(3):e18375. doi: 10.1371/journal.pone.0018375 (PMC3069090; doi:10.1371/journal.pone.0018375)
Supplement: Table S2 — The calculated epistasis values and statistics for the RTase 2-point mutants along an AZT resistance pathway under different AZT concentrations in the TZM-bl cell line (a), Donor 1 (b) and Donor 2 (c). Calculations were made according to equation 1 (see main text and materials and methods for details). (DOC) [file pone.0018375.s003.doc]

**Table S2a.** Epistasis values and statistics for the AZT-resistant HIV-1 RTase double mutants in the TZM-bl cell line

|  |  | **Variants** | | | | | |
| --- | --- | --- | --- | --- | --- | --- | --- |
|  |  | **General formulas** | | | **Bootstrap analysis** | | |
| **AZT (µM)** | **Statistics** | **M41L/T215N** | **M41L/T215S** | **M41L/T215Y** | **M41L/T215N** | **M41L/T215S** | **M41L/T215Y** |
| **0** | **mean** | **0,10087** | **0,1098** | **0,31576** | **1,01E-01** | **1,08E-01** | **3,15E-01** |
|  | **var** | 4,20E-03 | 5,67E-03 | 2,75E-02 | 3,83E-03 | 5,95E-03 | 2,76E-02 |
|  | **std.dev.** | 0,06481 | 0,075269 | 0,16573 | 6,19E-02 | 7,71E-02 | 1,66E-01 |
|  | **std.err.** | 0,032405 | 0,0376345 | 0,082865 | 1,96E-03 | 2,44E-03 | 5,26E-03 |
| **0.03** | **mean** | **-0,0075061** | **-0,01581** | **-0,028959** | **-7,84E-03** | **-1,62E-02** | **-3,34E-02** |
|  | **var** | 8,75E-04 | 1,02E-03 | 7,14E-03 | 9,59E-04 | 1,06E-03 | 7,72E-03 |
|  | **std.dev.** | 0,029575 | 0,031958 | 0,0845 | 3,10E-02 | 3,26E-02 | 8,79E-02 |
|  | **std.err.** | 0,0147875 | 0,015979 | 0,04225 | 9,79E-04 | 1,03E-03 | 2,78E-03 |
| **0.3** | **mean** | **-0,019783** | **-0,01857** | **-0,097025** | **-0,019524** | **-0,018497** | **-9,59E-02** |
|  | **var** | 3,68E-05 | 4,50E-05 | 8,47E-04 | 3,51E-05 | 4,47E-05 | 8,68E-04 |
|  | **std.dev** | 0,0060643 | 0,0067053 | 0,029096 | 5,92E-03 | 6,69E-03 | 2,95E-02 |
|  | **std.err.** | 0,00303215 | 0,00335265 | 0,014548 | 1,87E-04 | 2,11E-04 | 9,32E-04 |
| **2** | **mean** | **-0,0010159** | **-0,00108** | **-0,014587** | **-0,0010133** | **-0,0010799** | **-0,014619** |
|  | **var** | 1,30E-07 | 8,27E-08 | 1,64E-05 | 1,21E-07 | 8,72E-08 | 1,73E-05 |
|  | **std.dev.** | 0,00036064 | 0,00028759 | 0,0040488 | 3,47E-04 | 2,95E-04 | 4,16E-03 |
|  | **std.err.** | 0,00018032 | 0,000143795 | 0,0020244 | 1,10E-05 | 9,34E-06 | 1,31E-04 |
| **5** | **mean** | **-1,45E-04** | **-1,78E-04** | **-0,004138** | **-1,45E-04** | **-1,75E-04** | **-4,17E-03** |
|  | **var** | 2,49E-09 | 3,63E-09 | 1,18E-06 | 2,60E-09 | 3,66E-09 | 1,17E-06 |
|  | **std.dev** | 4,99E-05 | 6,02E-05 | 0,0010842 | 5,10E-05 | 6,05E-05 | 1,08E-03 |
|  | **std.err.** | 2,49705E-05 | 0,00003012 | 0,0005421 | 1,61E-06 | 1,91E-06 | 3,42E-05 |
| **10** | **mean** | **-1,55E-05** | **-2,43E-05** | **-9,15E-04** | **-1,54E-05** | **-2,41E-05** | **-9,19E-04** |
|  | **var** | 7,24E-11 | 8,61E-11 | 8,45E-08 | 7,76E-11 | 8,56E-11 | 8,53E-08 |
|  | **std.dev.** | 8,51E-06 | 9,28E-06 | 2,91E-04 | 8,81E-06 | 9,25E-06 | 2,92E-04 |
|  | **std.err.** | 4,25495E-06 | 4,6401E-06 | 0,000145305 | 2,79E-07 | 2,93E-07 | 9,24E-06 |

**Table S2b.** Epistasis values and statistics for the AZT-resistant HIV-1 RTase double mutants in PBMC from Donor 1.

|  |  | **Variants** | | | | | |
| --- | --- | --- | --- | --- | --- | --- | --- |
|  |  | **General formulas** | | | **Bootstrap analysis** | | |
| **AZT (µM)** | **Statistics** | **M41L/T215N** | **M41L/T215S** | **M41L/T215Y** | **M41L/T215N** | **M41L/T215S** | **M41L/T215Y** |
| **0** | **mean** | **0,13269** | **0,28286** | **0,19914** | **0,13412** | **0,28663** | **0,20088** |
|  | **var** | 3,79E-03 | 8,34E-03 | 1,11E-02 | 0,0036157 | 0,0097278 | 0,011481 |
|  | **std.dev.** | 0,061561 | 0,091299 | 0,1053 | 0,060131 | 0,09863 | 0,10715 |
|  | **std.err.** | 0,0307805 | 0,0456495 | 0,05265 | 0,0019015 | 0,0031189 | 0,0033884 |
| **0.03** | **mean** | **0,01379** | **0,11306** | **0,0613** | **0,013079** | **0,11456** | **0,05985** |
|  | **var** | 7,95E-04 | 1,83E-03 | 8,77E-03 | 0,00082894 | 0,0018623 | 0,009005 |
|  | **std.dev.** | 0,028198 | 0,042831 | 0,093632 | 0,028791 | 0,043154 | 0,094895 |
|  | **std.err.** | 0,014099 | 0,0214155 | 0,046816 | 0,00091046 | 0,0013647 | 0,0030008 |
| **0.3** | **mean** | **0,0018382** | **0,017584** | **-0,0064763** | **0,0018504** | **0,017555** | **-0,0054002** |
|  | **var** | 4,22E-06 | 3,50E-05 | 4,96E-04 | 4,14E-06 | 3,33E-05 | 0,00049241 |
|  | **std.dev** | 0,0020538 | 0,0059167 | 0,02226 | 0,0020348 | 0,0057677 | 0,02219 |
|  | **std.err.** | 0,0010269 | 0,00295835 | 0,01113 | 6,43E-05 | 0,00018239 | 0,00070172 |
| **2** | **mean** | **Not Applicable** | **4,60E-05** | **-0,0066982** | **NA** | **5,01E-05** | **-0,0068068** |
|  | **var** | **(NA)** | 2,56E-07 | 3,49E-05 |  | 2,61E-07 | 3,64E-05 |
|  | **std.dev.** |  | 0,00050573 | 0,0059083 |  | 0,00051072 | 0,0060318 |
|  | **std.err.** |  | 0,000252865 | 0,00295415 |  | 1,62E-05 | 0,00019074 |
| **5** | **mean** | **NA** | **NA** | **-0,0011412** | **NA** | **NA** | **-0,0011527** |
|  | **var** |  |  | 2,10E-06 |  |  | 1,99E-06 |
|  | **std.dev** |  |  | 0,0014503 |  |  | 0,0014113 |
|  | **std.err.** |  |  | 0,00072515 |  |  | 4,46E-05 |
| **10** | **mean** | **NA** | **NA** | **NA** | **NA** | **NA** | **NA** |
|  | **var** |  |  |  |  |  |  |
|  | **std.dev.** |  |  |  |  |  |  |
|  | **std.err.** |  |  |  |  |  |  |

**Table S2c.** Epistasis values and statistics for the AZT-resistant HIV-1 RTase double mutants in PBMC from Donor 2.

|  |  | **Variants** | | | | | |
| --- | --- | --- | --- | --- | --- | --- | --- |
|  |  | **General formulas** | | | **Bootstrap analysis** | | |
| **AZT (µM)** |  | **M41L/T215N** | **M41L/T215S** | **M41L/T215Y** | **M41L/T215N** | **M41L/T215S** | **M41L/T215Y** |
| **0** | **mean** | **0,16856** | **0,34031** | **0,35699** | **1,65E-01** | **3,37E-01** | **3,60E-01** |
|  | **var** | 5,16E-03 | 1,11E-02 | 1,68E-02 | 5,00E-03 | 1,04E-02 | 1,75E-02 |
|  | **std.dev.** | 0,07186 | 0,10531 | 0,12952 | 7,07E-02 | 1,02E-01 | 1,32E-01 |
|  | **std.err.** | 0,03593 | 0,052655 | 0,06476 | 2,24E-03 | 3,23E-03 | 4,18E-03 |
| **0.03** | **mean** | **0,020853** | **0,16906** | **0,037667** | **2,10E-02** | **1,68E-01** | **4,23E-02** |
|  | **var** | 3,35E-04 | 1,42E-03 | 6,60E-03 | 3,43E-04 | 1,46E-03 | 6,48E-03 |
|  | **std.dev.** | 0,018292 | 0,03763 | 0,081238 | 1,85E-02 | 3,82E-02 | 8,05E-02 |
|  | **std.err.** | 0,009146 | 0,018815 | 0,040619 | 5,86E-04 | 1,21E-03 | 2,55E-03 |
| **0.3** | **mean** | **0,0041938** | **0,016181** | **0,013825** | **0,0043255** | **0,016194** | **1,48E-02** |
|  | **var** | 5,88E-06 | 1,12E-05 | 3,98E-04 | 5,93E-06 | 1,17E-05 | 4,35E-04 |
|  | **std.dev** | 0,0024255 | 0,0033519 | 0,019954 | 2,43E-03 | 3,42E-03 | 2,09E-02 |
|  | **std.err.** | 0,00121275 | 0,00167595 | 0,009977 | 7,70E-05 | 1,08E-04 | 6,60E-04 |
| **2** | **mean** | **0,00083403** | **0,00030326** | **0,005245** | **0,00084001** | **0,00030908** | **0,0052025** |
|  | **var** | 5,08E-08 | 1,17E-08 | 2,42E-06 | 4,99E-08 | 1,19E-08 | 2,53E-06 |
|  | **std.dev.** | 0,00022539 | 0,00010837 | 0,001555 | 2,23E-04 | 1,09E-04 | 1,59E-03 |
|  | **std.err.** | 0,000112695 | 0,000054185 | 0,0007775 | 7,06E-06 | 3,46E-06 | 5,03E-05 |
| **5** | **mean** | **NA** | **NA** | **0,00019382** | **NA** | **NA** | **2,06E-04** |
|  | **var** |  |  | 3,60E-07 |  |  | 3,74E-07 |
|  | **std.dev** |  |  | 0,00060003 |  |  | 6,12E-04 |
|  | **std.err.** |  |  | 0,000300015 |  |  | 1,93E-05 |
| **10** | **mean** | **NA** | **NA** | **NA** | **NA** | **NA** | **NA** |
|  | **var** |  |  |  |  |  |  |
|  | **std.dev.** |  |  |  |  |  |  |
|  | **std.err.** |  |  |  |  |  |  |
|  |  |  |  |  |  |  |  |
