# Supplementary material for: Fitness Ranking of Individual Mutants Drives Patterns of Epistatic Interactions in HIV-1
Source: PLoS One. 2011 Mar 31;6(3):e18375. doi: 10.1371/journal.pone.0018375 (PMC3069090; doi:10.1371/journal.pone.0018375)
Supplement: Table S3 — Relative frequency values for the RTase 2-point mutants along an AZT resistance pathway under different AZT concentrations in the TZM-bl cell line (a), Donor 1 (b) and Donor 2 (c). Relative frequencies with positive epistasis are noted in bold and with negative epistasis in italic. Frequencies were calculated according to equation 2 (see main text for details). (DOC) [file pone.0018375.s004.doc]

**Table S3a.** Relative frequency values for the 2-point AZT-resistant HIV-1 RTase mutants in the TZM-bl cell line for Positive Epistasis (bold) and Negative Epistasis (italic).

| **AZT (µM)** | **M41L/T215N**  **Epistasis**  **(No epistasis)** | **M41L/T215S**  **Epistasis**  **(No epistasis)** | **M41L/T215Y Epistasis**  **(No epistasis)** |
| --- | --- | --- | --- |
| **0** | **4.9*10^-9^**  (4.3*10^-9^) | **5.3*10^-9^**  (4.7*10^-9^) | **1.4*10^-8^**  (7.8*10^-9^) |
| **0.03** | *6.34*10^-5^*  (6.72*10^-5^) | *7.07*10^-5^*  (8.16*10^-5^) | *5.5*10^-4^*  (9.99*10^-1^) |
| **0.3** | *5.78*10^-5^*  (9.999*10^-1^) | *6.92*10^-5^*  (9.999*10^-1^) | *3.42*10^-4^*  (9.999*10^-1^) |
| **2** | *5.25*10^-5^*  (9.999*10^-1^) | *5.84*10^-5^*  (9.999*10^-1^) | *9.998*10^-1^*  (9.999*10^-1^) |
| **5** | *4.95*10^-5^*  (9.999*10^-1^) | *6.0*10^-5^*  (9.999*10^-1^) | *9.998*10^-1^*  (9.999*10^-1^) |
| **10** | *5.33*10^-5^*  (9.999*10^-1^) | *5.72*10^-5^*  (9.999*10^-1^) | *9.999*10^-1^*  (9.9992*10^-1^) |

**Table S3b.** Relative frequency values for 2-point AZT-resistant HIV-1 RTase mutants in PBMC from Donor 1 for Positive Epistasis (bold) and Negative Epistasis (italic)

| **AZT (µM)** | **M41L/T215N**  **Epistasis**  **(No epistasis)** | **M41L/T215S**  **Epistasis**  **(No epistasis)** | **M41L/T215Y**  **Epistasis**  **(No epistasis)** |
| --- | --- | --- | --- |
| **0** | **6.4*10^-9^**  (5.3*10^-9^) | **8.6*10^-9^**  (5.6*10^-9^) | **1.1*10^-8^**  (7.6*10^-9^) |
| **0.03** | **3.1*10^-8^**  (2.9*10^-8^) | **5.2*10^-8^**  (3.0*10^-8^) | **1.9*10^-7^**  (8.4*10^-8^) |
| **0.3** | **4.4*10^-5^**  (4.2*10^-5^) | **7.8*10^-5^**  (4.4*10^-5^) | *9.995*10^-1^*  (9.997*10^-1^) |
| **2** | **Not Applicable (NA)** | **4.4*10^-5^**  (4.3*10^-5^) | *9.997*10^-1^*  (9.999*10^-1^) |
| **5** | NA | NA | *9.998*10^-1^*  (9.999*10^-1^) |
| **10** | NA | NA | NA |

**Table S3c.** Relative frequency values for 2-point AZT-resistant HIV-1 RTase mutants in PBMC from Donor 2 for Positive Epistasis (bold) and Negative Epistasis (regular)

| **AZT (µM)** | **M41L/T215N**  **Epistasis**  **(No epistasis)** | **M41L/T215S**  **Epistasis**  **(No epistasis)** | **M41L/T215Y**  **Epistasis**  **(No epistasis)** |
| --- | --- | --- | --- |
| **0** | **6.3*10^-9^**  (5.1*10^-9^) | **9.0*10^-9^**  (5.4*10^-9^) | **3.6*10^-8^**  (1.3*10^-8^) |
| **0.03** | **2.3*10^-8^**  (2.1*10^-8^) | **6.0*10^-8^**  (2.1*10^-8^) | **0.998**  (4.4*10^-4^) |
| **0.3** | **5.2*10^-5^**  (4.3*10^-5^) | **1.3*10^-4^**  (4.4*10^-5^) | **0.999**  (0.998) |
| **2** | **6.9*10^-9^**  (3.7*10^-9^) | **4.8*10^-9^**  (3.9*10^-9^) | **0.9999**  (8.97*10^-5^) |
| **5** | NA | NA | **0.9999**  (0.99988) |
| **10** | NA | NA | NA |
