# Supplementary material for: Fitness Ranking of Individual Mutants Drives Patterns of Epistatic Interactions in HIV-1
Source: PLoS One. 2011 Mar 31;6(3):e18375. doi: 10.1371/journal.pone.0018375 (PMC3069090; doi:10.1371/journal.pone.0018375)
Supplement: Table S4 — Evaluation of statistical significance of the differences in mean relative fitness between wild type and AZT-resistant variants for Donor 2 by a Student-Newman-Keuls test. Fitness values are taken from Table S1c. Calculations were performed in Graphpad Prism. The examples selected here for statistical evaluation are discussed under results. (DOC) [file pone.0018375.s005.doc]

**Table S4.** Evaluation of statistical significance of the differences in mean relative fitness between wild type and AZT-resistant variants for Donor 2 by a Student-Newman-Keuls test. Fitness values are taken from Table S1c. The examples selected here for statistical evaluation are discussed under results.

**Donor 2**

**0.3 µM AZT**

Comparison Difference P value

=============== ======== ==========

WT vs M41L -0.004010 ns P>0.05

WT vs T215Y -0.1303 * P<0.05

M41L vs T215Y -0.1262 ** P<0.01

WT vs M41L/T215Y -0.2227 *** P<0.001

M41L vs M41L/T215Y -0.2187 *** P<0.001

T215Y vs M41L/T215Y -0.09244 * P<0.05

WT vs M41L/T215Y (no) -0.1375 *** P<0.001

M41L vs M41L/T215Y (no) -0.1335 *** P<0.001

T215Y vs M41L/T215Y (no) -0.007210 ns P>0.05

*****, level of significance; **ns**, non significant; (ep), experimental; (no), no epistasis

**2 µM AZT**

Comparison Difference P value

=============== ======== ==========

WT vs M41L -0.01901 ns P>0.05

WT vs T215Y -0.07343 *** P<0.001

M41L vs T215Y -0.09244 *** P<0.001

WT vs M41L/T215Y -0.1447 *** P<0.001

M41L vs M41L/T215Y -0.1637 *** P<0.001

T215Y vs M41L/T215Y -0.07124 *** P<0.001

WT vs M41L/T215Y (no) -0.02168 ** P<0.01

M41L vs M41L/T215Y (no) -0.04069 *** P<0.001

T215Y vs M41L/T215Y (no) -0.05175 *** P<0.001

*****, level of significance; **ns**, non significant; (ep), experimental; (no), no epistasis
