# Supplementary material for: Fitness Ranking of Individual Mutants Drives Patterns of Epistatic Interactions in HIV-1
Source: PLoS One. 2011 Mar 31;6(3):e18375. doi: 10.1371/journal.pone.0018375 (PMC3069090; doi:10.1371/journal.pone.0018375)
Supplement: Table S5 — Primers uses for the site-directed PCR to generate the HIV-1 RTase mutants used in this study. Restriction sites are underlined and bold, codons with the introduced mutantions are underlined. (DOC) [file pone.0018375.s006.doc]

**Table S5.** Primers used in the site-directed PCR to generate the HIV-1 RTase mutants.

| **Substitution** | **Template** | **Primer Name** | **Sequence (1)** |
| --- | --- | --- | --- |
| - | NL4-3 | 3'AgeI | **accggt**tcttttagaatctccctgttt |
| - | NL4-3 | Becl 5' | **tgatca**gatactcatagaaatctgcgg |
| M41L | NL4-3 | 41L-f | gaaatttgtacagaattggaaaaggaaggaaaaatttc |
|  | NL4-3 | 41L-r | gaaatttttccttccttttccaattctgtacaaatttc |
| T215N | NL4-3 | 215N-f | gtggggatttaacacaccagac |
|  | NL4-3 | 215N-r | gtctggtgtgttaaatccccac |
| T215S | NL4-3 | 215S-f | gtggggattttccacaccagac |
|  | NL4-3 | 215S-r | gtctggtctccaaaatccccac |
| T215Y | NL4-3 | 215Y-f | gtggggattttacacaccagac |
|  | NL4-3 | 215Y-r | gtctggtgtgtaaaatccccac |

(1): Underlined and bold: restriction sites; underlined: codons with the introduced mutations
